# Small intestinal strangulation in 60 cattle – clinical, laboratory and ultrasonographic findings, treatment and outcome

**DOI:** 10.1186/s12917-023-03797-9

**Published:** 2023-11-09

**Authors:** Ueli Braun, Christian Gerspach, Sandra Loss, Monika Hilbe, Karl Nuss

**Affiliations:** 1 Department of Farm Animals, Vetsuisse Faculty, University of Zurich, Winterthurerstrasse 260, Zurich, CH-8057 Switzerland; 2 Institute of Veterinary Pathology, Vetsuisse Faculty, University of Zurich, Winterthurerstrasse 260, Zurich, CH-8057 Switzerland

**Keywords:** Cattle, Small intestine, Ileus, Mechanical obstruction, Strangulation

## Abstract

**Background:**

Intestinal strangulation is constriction of the intestine by a band of tissue, ligament or blood vessel causing partial or complete intestinal obstruction. This retrospective study describes the clinical, laboratory and ultrasonographic findings, treatment and outcome of 60 cows with intestinal strangulation.

**Results:**

The general condition was abnormal in all cows (60/60), 23.3% (14/60) had nonspecific signs of pain, 40.0% (24/60) had signs of colic and 48.3% (29/60) had signs of somatic (parietal) pain. The most common digestive tract abnormalities were, in decreasing frequency, reduced or absent intestinal motility (100%, 60/60), reduced or absent faecal output (98.3%, 59/60), reduced or absent rumen motility (93.4%, 56/60), dilated small intestines on transrectal palpation (63.3%, 38/60), positive ballottement and swinging auscultation (BSA) and/or percussion and simultaneous auscultation (PSA) on the right side of the abdomen (58.3%, 35/60) and at least one positive foreign body test, most commonly the back grip, in 33.9% (20/59) of the cows. Other common findings were reduced skin surface temperature (67.8%, 40/59), reduced skin turgor (51.7%, 31/60), prolonged capillary refill time (49.2%, 29/59), enophthalmus (48.3%, 29/60) and moderate to severe scleral injection (46.6%, 27/58). The most common laboratory findings were hypokalaemia (58.3%, 35/60), haemoconcentration (57.6%, 34/59), base excess (51.1%, 24/47), hyperproteinaemia (45.8%, 27/59), hyperbilirubinaemia (43.3%, 26/60), acidosis (42.6%, 20/47) and azotaemia (38.3%, 23/60). The principal ultrasonographic findings were subjectively reduced or absent small intestinal motility and dilated small intestines, but the strangulation could not be visualised by ultrasonography. With one exception, all cows underwent a right flank laparotomy to resolve the strangulation by transection or resection of the impinging tissue. Forty-nine (81.7%) cows were discharged and 11 (18.3%) were euthanized before, during or after surgery.

**Conclusions:**

Without laparotomy, intestinal strangulation could be clinically (transrectally) diagnosed in only 10% of the cows. A laparotomy is therefore essential for the correct diagnosis. The prognosis is good with prompt surgical treatment.

## Background

Intestinal strangulation is an impingement of the intestine by tissue bands of various origins that extend between two abdominal organs or between an organ and the abdominal wall or are freely floating in the abdominal cavity, causing a partial or complete intestinal obstruction [1]. Intestinal strangulation has rarely been described in cattle; of 27 cases of small intestinal obstruction in cows, only three (11%) were caused by strangulation [2]. The tissue bands may consist of connective tissue, ligaments or blood vessels. The formation of connective or fibroserosal tissue bands is often preceded by peritonitis caused by fascioliasis, perimetritis, hardware disease, abdominal surgery, intraperitoneal injections or other peritoneal irritation [1]. Pedunculated lipomas, ligamentous anomalies or incomplete involution of the umbilicus or urachal structures are other causes of intestinal strangulation [1, 3–7]. A condition referred to as gut tie has been described in male cattle, in which the recoiling ductus deferens causes intestinal strangulation after open castration [1, 8, 9]. A persistent 30-cm vitelloumbilical band was the cause of small intestinal strangulation (SIS) in a Holstein cow [3] and a persistent round ligament running caudoventrally from the visceral surface of the liver to the umbilicus caused SIS in two cows [4, 7]. Persistent urachal remnants were responsible for SIS in a two-year-old Beefmaster cow [6] and in a three-year-old Brown Swiss cow [10]. Another report involved a one-month-old Hereford calf with omphaloarteritis, in which a band from the greater omentum to the left umbilical artery caused strangulation of the jejunum [5].

The clinical manifestation of SIS depends on the extent, degree and anatomic location of the constriction [1]. Provided that obstruction of the intestinal lumen is merely partial or occurs only periodically, the disease course can be protracted over several days during which time the patient shows signs of persistent or recurring colic with reduced but not necessarily absent defaecation [1]. In cattle with complete intestinal obstruction, rectal examination may reveal the absence of faecal material with only mucoid contents. Dilated intestinal loops, and sometimes a taut band, may be palpated transrectally [1]. The principal ultrasonographic findings in cattle with ileus are reduced or absent intestinal motility and dilated small intestines [11, 12]. To our knowledge, ultrasonographic visualisation of intestinal strangulation has not been reported.

Treatment of intestinal strangulation is surgical [1, 13] and right flank laparotomy is the surgical approach of choice. In most cases, the strangulation can be identified and rectified using surgical scissors, tenotomy knives or blunt methods to cut the band or bands. Ideally, the severed structures are resected. Intestinal viability is assessed, and non-viable segments are resected if necessary [1]. Two Brown Swiss cows with prolapse of the pregnant uterus through a defect in the mesoduodenum causing strangulation of the duodenum required alternative treatment because retraction of the enlarged uterus through the mesoduodenal defect was not possible. Transection and end-to-end anastomosis of the duodenum was required to treat the strangulation [14]. As in other types of ileus, the cornerstones of postoperative treatment include intravenous fluids, antibiotics, analgesics and electrolyte replacement.

Even though most veterinary textbooks cover SIS, large numbers of cattle with the disorder have not been analysed. The above-mentioned publications describe 1 to a maximum of 3 cases. In addition, the published cases underwent only clinical and laboratory analyses before laparotomy. In only one case was ultrasonography carried out [14]. Therefore, the goal of the present study was to further our knowledge by describing the clinical, laboratory and ultrasonographic findings, treatment, and outcome of 60 cattle with SIS.

## Methods

All methods were performed in accordance with relevant guidelines and regulations.

As part of a Master’s thesis [15], the medical records of 60 cattle referred to the Department of Farm Animals, University of Zurich, between January 1, 1990 and December 31, 2019, with SIS were analysed. Thirty-two cows (53.3%) had shown signs of colic, which led to a tentative diagnosis of mechanical obstruction of the small intestines. Caecal dilatation and/or torsion was the tentative diagnosis in six other cows, right displaced abomasum in two and traumatic reticuloperitonitis in one cow. Nineteen cows were referred without a tentative diagnosis.

### Inclusion and exclusion criteria

For inclusion in the study, only the records of cows that were a minimum of one year of age and had SIS at the time of admission were reviewed. The diagnosis of SIS had to have been confirmed unequivocally during laparotomy or postmortem examination. Two records were excluded from analysis because they had been published previously [14]. Another five of the 65 records included in the Master’s thesis [15] were excluded because they did not fulfil the definition of strangulation (intestinal occlusion by a band of connective tissue, ligament or blood vessel).

### Animals

There were 51 mature cows (85.0%) and 9 heifers (young cows that have not yet given birth to a calf, 15.0%) that were between 1.1 and 11 years of age (median = 4 years). For the purpose of this report, all were referred to as cows. Breeds included Swiss Braunvieh (75.0%, 45/60), Holstein (13.3%, 8/60), Swiss Fleckvieh (10.0%, 6/60) and Hereford (1.7%, 1/60). Of the cows, 45.0% (27/60) were pregnant (from 6 to 41 weeks, median = 41 weeks), 28.3% (17/60) were open, and in the remaining 26.7% (16/60), the pregnancy status was not known at the time of admission. The last calving date was known for 25 cows and was between 1 and 26 weeks before admission (mean ± sd = 11.3 ± 7.6 weeks). Nine (15.0%) animals had never calved. The duration of illness before admission ranged from 4 to 120 h (median = 24 h). A history of complete anorexia occurred in 68.3% (41/60) of the cows and 31.7% (19/60) had a reduced appetite. Thirty-two (53.3%) had a history of colic before admission.

### Clinical examination

All cows underwent the same structured clinical examination procedure conducted by the first author or under the supervision of the first or second author. Clinical examination was done according to published standards [16–18] and as described recently in detail [18]. General condition was evaluated by determining the demeanour, the appearance of the hair coat and muzzle, the skin elasticity, the position of the eyes in the sockets and skin surface temperature. General condition was classified as normal or mildly, moderately or severely abnormal. Cows with a normal general condition were bright and alert and had normal behaviour, posture and appetite. The general condition was considered mildly abnormal when a mild decrease in alertness and/or mild signs of colic (defined below) were present. A moderate decrease in alertness and sometimes occasional grunting, and/or bruxism and marked signs of colic were observed in cattle with a moderately abnormal general condition. Cattle with a severely abnormal general condition showed marked apathy and were sometimes recumbent and unable to rise. The rumen was assessed for the degree of fill and the number and intensity of contractions. Sensitivity in the reticular region was assessed by preventing the animal from breathing for a short period by placing a plastic rectal sleeve over the mouth and nose and listening for grunting during the ensuing deep breath. This was followed by foreign body tests, which included the pole test, back grip and percussion of the abdominal wall over the region of the reticulum using a rubber hammer. Each test was carried out four times, and the reaction of the animal was observed each time. A test was considered positive when it elicited a short grunt a minimum of three of four times. The response to a test was considered questionable when it elicited a grunt two of four times and negative when the animal did not grunt or grunted only once. Ballottement and simultaneous auscultation (BSA) as well as percussion and simultaneous auscultation (PSA) of the abdomen on both sides were also carried out. BSA was considered positive when splashing sounds were heard with a stethoscope while the abdominal wall was manually ballotted to produce a swinging motion. PSA was considered positive when a ringing sound or ping was heard on percussion of the abdominal wall with the handle of a hammer. Rectal examination was done in all cows. Faeces were assessed for colour, consistency, amount, fibre particle length and abnormal contents.

Each cow was observed for signs of pain. Signs of pain were divided into non-specific, somatic (parietal) and visceral (colic, abdominal pain). Non-specific signs of pain included muscle fasciculations, bruxism and spontaneous grunting. Signs of somatic (parietal) pain were a tense abdominal wall, arching of the back and a tucked-up abdomen. Visceral (colic, abdominal pain) signs consisted of shifting of weight in the hind limbs, lordosis, restlessness, kicking at the abdomen, sweating, tail swishing, frequent lying down and rising. The number and severity of signs of colic/abdominal pain were determined. Signs of mild colic included mild restlessness, shifting of weight in the hind limbs, looking at the flank, lifting the tail, lifting of individual limbs and tail swishing. Signs of moderate colic were moderate restlessness, brief periods of recumbency, kicking with the hind limbs, arching of the back and marked tail swishing. Signs of severe colic consisted of marked restlessness, frequent lying down and rising, sweating, grunting and violent kicking at the abdomen.

### Laboratory analyses

The following blood samples were collected from all cattle: 5 ml of EDTA blood for haematological analysis, 10 ml of whole blood for serum biochemistry and 2 ml of whole blood mixed with 0.2 ml heparin for venous blood gas analysis. The variables were measured as described previously [17]. Haematological analysis included the determination of haematocrit, total leukocyte count and the concentrations of total protein and fibrinogen. The samples were analysed using the Contraves analyzer AL820 (Contraves, Oerlikon, Switzerland) or the CELL-DYN 3500 (Abbott Diagnostics Division, Baar, Switzerland). The concentrations of urea, bilirubin, calcium, magnesium, inorganic phosphate, chloride, potassium, and the activities of the enzymes aspartate aminotransferase (AST) and γ-glutamyltransferase (γ-GT) were determined at 37 °C using an automated analyzer (Cobas Mira, Cobas Integra 700, Cobas Integra 800, Roche Diagnostics, Basel, Switzerland) and the manufacturer’s reagents (Roche-Reagents) according to the International Federation of Clinical Chemistry and Laboratory Medicine (IFCC). The venous blood gas analysis was performed with the RapidLab 248 analyser (Siemens Schweiz AG, Zurich, Switzerland).

Urine samples were analysed using a test strip (Combur^9^, Roche, Basel, Switzerland) and a refractometer (Krüss Optronic, Hamburg, Germany) to measure specific gravity. A sample of rumen fluid was collected using a Dirksen probe and assessed for colour, odour, consistency and pH (data not shown). In addition, the concentration of chloride was determined (MK-II-Chlorid-Analyser 9265, Sherwood, Cambridge, Great Britain).

### Parasitological analysis of faecal samples

The faeces of 25 cattle were examined for *Fasciola hepatica*, *Dicrocoelium dendriticum*, gastrointestinal strongyles and *Dictyocaulus viviparus* at the Institute for Parasitology, Vetsuisse Faculty, University of Zurich.

### Ultrasonographic examination of the abdomen

Forty-three cattle underwent ultrasonographic examination of the right side of the abdomen [11]. Briefly, the area from the tuber coxae to the eighth intercostal space and from the transverse processes of the vertebrae to the linea alba on the right side was examined using a 5.0 MHz linear or convex transducer. The appearance of loops of small intestine and their diameter, contents and motility were assessed. In addition, the appearance, position and nature of the contents of the caecum and proximal and spiral ansa of the colon and the presence of caecal dilatation were noted.

### Diagnosis

A *tentative clinical diagnosis of ileus* was made in cattle with a history of abdominal pain and in cattle that had signs of abdominal pain and little or no manure in the rectum at the initial examination. A *diagnosis of ileus* was made when dilated loops of small intestines could be palpated transrectally. A *tentative diagnosis of ileus attributable to strangulation* was made when in addition to the aforementioned findings a taut tissue band was palpated transrectally.

An *ultrasonographic diagnosis of ileus* was made when dilated small intestines with a diameter exceeding 3.5 cm and minimal or no motility were seen accompanied by the aforementioned clinical signs. The final diagnosis was made during laparotomy and/or at postmortem examination.

### Laparotomy

All but 1 cow underwent right-flank laparotomy. Before 2001, distal paravertebral anaesthesia of the last thoracic and first two lumbar spinal nerves was done using lidocaine as described [19, 20]. Proximal paravertebral anaesthesia of the same nerves was then carried out starting in 2001. A vertical incision through all layers of the abdominal wall was made in the centre of the paralumbar fossa, starting 7–10 cm below the transverse processes and extending about 25 cm distally. In 1 cow with an asternal rib, the incision was made near the caudal border of the paralumbar fossa, and in another cow with strangulation of the duodenum, the last rib was resected to allow for an incision cranial to the paralumbar fossa. After routine abdominal exploration, identified tissue bands were severed by blunt or sharp dissection, ligated and transected, or the strangulation was resolved during exteriorisation of the intestines. After the surgical procedure, an antibiotic, most commonly amoxicillin, was infused into the abdomen in 1 L of isotonic saline solution or polyvinylpyrrolidone. The peritoneum, fascia and transverse abdominal muscle, and the internal and external oblique muscle layers were closed separately using a simple continuous suture pattern (Polysorb® 2USP, atraumatic needle, Covidien-Medtronic, Minneapolis, USA). A continuous subcuticular suture (Polysorb 2.0 USP cutting needle, Covidien-Medtronic, Minneapolis, USA – a modified mattress suture pattern) was used to close the subcutaneous tissues, and metal clips (Appose™, ULC 35 W clips, 6.9 mm x 3.8 mm, Covidien-Medtronic, Minneapolis, USA) were used to close the skin.

### Postoperative treatment

After surgery, the cows were fasted for 1 day before gradually introducing hay. Postoperative treatment included antibiotics, continuous intravenous drip infusions, analgesics and electrolytes. Antibiotic treatment included penicillin G procaine (12,000 IU/kg body weight, Procacillin®, MSD Animal Health) given intramuscularly once daily for 2 to 6 days, in most cases for 3 or 4 days (44/54, median = 3 days). Fifty-four cows received a daily injection of a non-steroidal anti-inflammatory drug (53/54, flunixin meglumine, 1 mg/kg, Flunixine®, Biokema, Crissier; or Ketoprofen, 3 mg/kg, Rifen®, Streuli Pharma) or a pyrazolone preparation (1/54, metamizole, 35 mg/kg, Vetalgin®, MSD Animal Health) given intravenously once daily for 2 to 5 days (median = 3 days). Forty-nine cows received 10 to 15 L of a solution containing 50 g glucose and 9 g sodium chloride per litre daily for 2 to 7 days (median = 3 days) administered as a slow intravenous drip (20 ml/kg/day) via an indwelling jugular vein catheter (Abbocath-T 14 g, length 14 cm, Abbott AG, Baar). Fourteen cows with hypocalcaemia (calcium < 2.0 mmol/l) received 500 ml of 40% calcium borogluconate supplemented with 6% magnesium hypophosphite (15.65 g calcium gluconate and borogluconate, 9.85 g magnesium hypophosphite; Calcamyl-40MP, Graeub, Bern) intravenously for 1 to 2 days (mean = 1 day). Hypokalaemia (potassium < 4.0 mmol/l) was treated in 15 cows with daily oral doses of 60 to 100 g of potassium chloride until normokalaemia was achieved (1 to 2 days, median = 1 day). Cows with hypophosphataemia (inorganic phosphorus < 1.0 mmol/L) or hypomagnesaemia (magnesium < 0.7 mmol/L) were treated orally with monocalcium phosphate, sodium dihydrogen phosphate and/or magnesium oxide. Prokinetic drugs were used in 43 cows for a duration of 1 to 9 days (median, 4 days). Thirty-four cows received intramuscular metoclopramide (30 mg) usually given seven to nine times at eight-hour intervals intramuscularly before its use in farm animals was discontinued in Switzerland, and 7 received neostigmine (40–45 mg/day, Konstigmin®, Vetoquinol, Bern) administered via continuous intravenous drip infusion. Five cows received additional antiparasitic treatment for fascioliasis and gastrointestinal nematodes including netobimin (7.5 mg/kg, Hapadex®, MSD Animal Health) or nitroxinil (10 mg/kg, Dovenix®, Biokema, Crissier). Two cows received 1 to 3 L of rumen fluid collected from a clinically healthy cow, 2 cows received 20 to 40 g dry yeast and 1 cow received 2 L mineral oil, administered orally.

### Euthanasia/slaughter

When indicated, cattle were slaughtered at the slaughter facility of the Veterinary Hospital, and the meat used for zoo-animal feeding, or they were euthanized using pentobarbital (Esconarkon, Streuli Pharma, 80 mg/kg body weight administered intravenously).

### Postmortem examination

All cows that died or were euthanized underwent postmortem examination. Only the internal organs were inspected in slaughtered cows.

### Statistics

The program SPSS Statistics 25.0 (IBM Corp. 2017, USA) was used for analysis. Frequencies were determined for all variables, and the Shapiro-Wilk test was used to test the data for normality. Normal data are presented as means ± standard deviations and non-normal data as medians. The variables heart rate and rectal temperature over time (day 0 to day 7) were analysed using the general linear model choosing repeated measures (ANOVA with repeated measures) and replacing *polynomial contrasts* with *difference*. Means and standard deviations, medians and the 25th to 75th percentiles were calculated for the different anatomic locations of the strangulating lesion (duodenum, jejunum, ileum, jejunum and ileum) and differences in medians were analysed using the Kruskal-Wallis test. The outcomes after transection and resection of tissue bands were compared using Fisher’s exact test because case numbers were small. P values < 0.05 were considered significant.

## Results

### General condition, abdominal contour and signs of pain

The general condition was mildly abnormal in 18.3% (11/60), moderately abnormal in 63.3% (38/60) and severely abnormal in 18.3% (11/60) of the cows. Bilateral abdominal distension occurred in 21.7% (13/60) of the cows, and 3.3% (2/60) had unilateral right-sided abdominal distension. Non-specific signs of pain occurred in 23.3% (14/60) of the cows and included twitching of the anconeus muscles (10.0%, 6/10), bruxism (8.3%, 5/60) and grunting (5.0%, 3/60). Abdominal guarding (tensing of the abdominal wall muscles, detected by pressing on the abdominal wall on the right side) as an expression of somatic (parietal) pain was seen in 48.3% (29/60) of the cows. One cow (1.7%) had an arched back, and another had a tucked-up abdomen. Signs of visceral pain (colic) were seen in 40.0% (24/60) of the cows and manifested as lowering of the back (25.0%, 15/60), treading (11.7%, 7/60), restlessness (10.0%, 6/60), kicking at the abdomen (10.0%, 6/60), sweating (5.0%, 3/60) and frequent lying down and rising (1.7%, 1/60). Of the cows with visceral pain, 21.7% (13/60) had one sign, 8.3% (5/60) had two signs and 6.7% (4/60) had four signs. Visceral pain was judged to be mild (28.3%, 17/60), moderate (5.0%, 3/60) or severe (6.7%, 4/60).

### Heart and respiratory rates and rectal temperature

Of these vital signs, the most common abnormalities were tachycardia (36.6%, 22/60, range = 48–120 beats per min., normal range = 60–80 beats per min.), decreased rectal temperature (31.7%, 19/60, range = 37.5–39.6 °C, normal range = 38.5–39.0 °C) and tachypnoea (35.0%, 21/60, range = 15–60 breaths per min., normal range = 15–25 breaths per min.) (Table 1).

**Table 1:** Clinical findings in cows with small intestinal strangulation (means ± standard deviations, medians with 25th to 75th percentiles, frequency distributions)

Variable	Finding		Number of cattle	%
Heart rate	Normal (60–80)		34	56.7
(n = 60,	Decreased (48–59)		4	6.7
79 ± 15 bpm)	Mildly increased (81–100)		17	28.3
	Moderately increased (101–120)		5	8.3
Rectal temperature	Normal (38.5–39.0)		26	43.3
(n = 60,	Decreased (37.5–38.4)		19	31.7
38.6 ± 0.6 °C)	Mildly increased (39.1–39.5)		14	23.3
	Moderately increased (39.6)		1	1.7
Respiratory rate	Normal (15–25)		38	63.3
(n = 60, 24, 20–28 breaths per min)	Decreased (12) Increased (26–60)		1 21	1.7 35.0
Rumen motility (n = 60)	Normal (2 or 3 strong contractions per 2 min) Decreased Absent		4 30 26	6.6 50.0 43.4
Foreign body tests (n = 59)	All negative At least one test positive^1^		39 20	66.1 33.9
BSA and PSA on the left side (n = 60)	Both negative (normal) Only BSA positive		59 1	98.3 1.7
BSA and PSA on the right side (n = 60)	Both negative (normal) Only BSA positive Only PA positive Both tests positive		25 18 5 12	41.7 30.0 8.3 20.0
Intestinal motility (n = 60)	Reduced Absent		42 18	70.0 30.0
Rectal findings^2^ (n = 60)	Normal findings Dilated small intestines Dilated small and large intestines Empty loops of small intestines Taut tissue strands Rumen dilated Miscellaneous abnormal findings		11 34 4 10 6 9 10	18.3 56.6 6.7 16.7 10.0 15.0 16.7
Faeces, amount (n = 60)	Normal Faecal output reduced Empty rectum		1 23 36	1.7 38.3 60.0
Faeces, colour and abnormal contents (n = 60)	Normal (olive) Dark to black Mucus Blood Fibrin More than one abnormality (mucus, blood, fibrin)		21 6 13 10 4 6	35.0 10.0 21.6 16.7 6.7 10.0
Faeces, degree of comminution (n = 60)	Normal (well digestion) Moderately digested Empty rectum		17 7 36	28.3 11.7 60.0
Faeces, consistency (n = 60)	Normal Thick pulpy Thin pulpy Greasy to pasty Liquid Empty rectum		12 7 2 2 1 36	20.0 11.7 3.3 3.3 1.7 60.0

^1^ Positive: at least 3 of 4 attempts elicited a grunt

^2^ The total number was 84 (140.0%), because 24 cows had more than one abnormal finding

### Digestive tract abnormalities

**Fig. 1:**
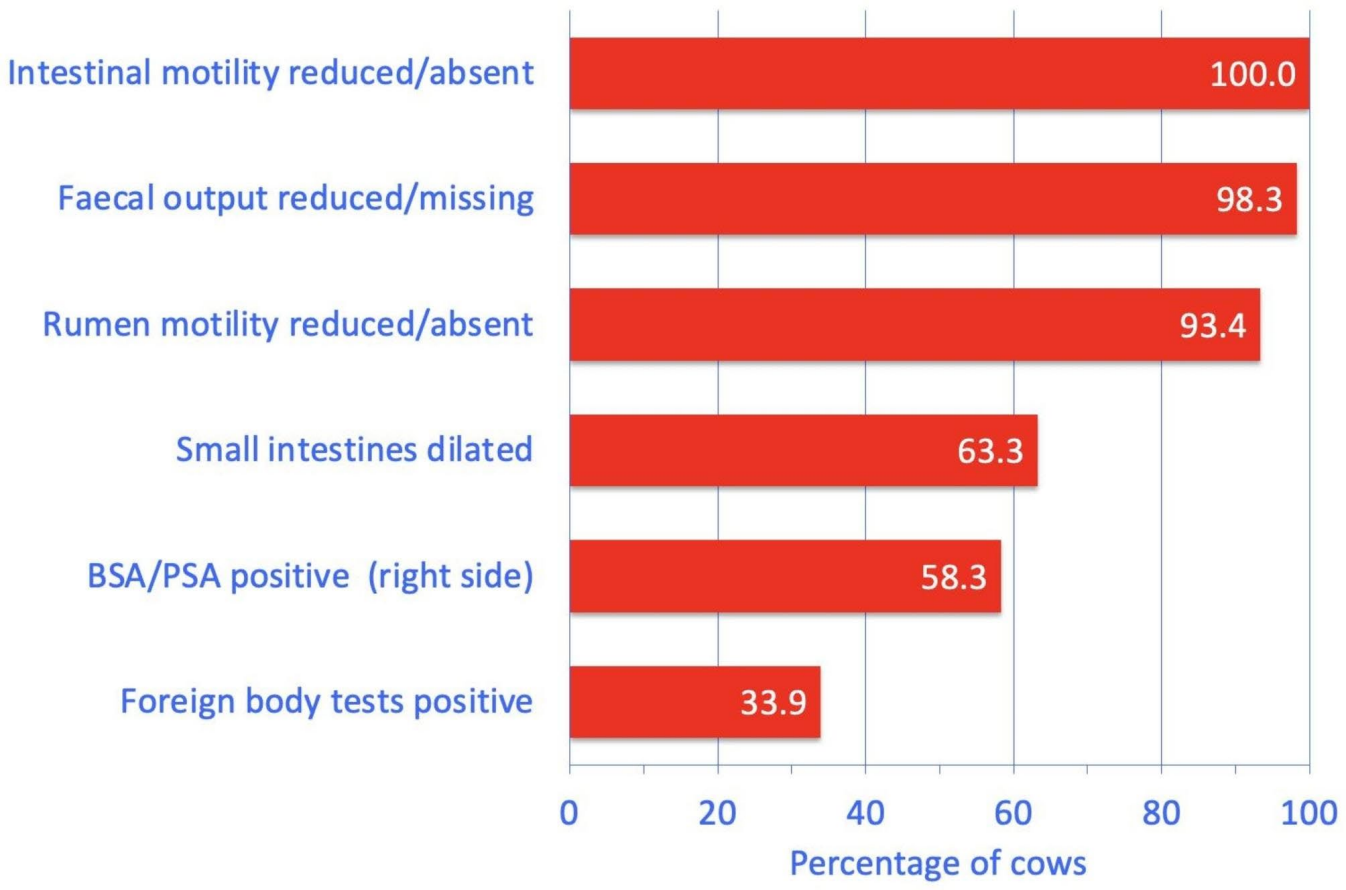
The most common digestive tract abnormalities found on clinical examination in 60 cows with small intestinal strangulation

The most common digestive tract abnormalities found on clinical examination were, in decreasing frequency, reduced or absent intestinal motility (100%, 60/60), reduced or absent faecal output (98.3%, 59/60), reduced or absent rumen motility (93.4%, 56/60, normal rate of rumen sounds = 2 or 3 strong contractions per 2 min.), dilated small intestines on transrectal palpation (63.3%, 38/60), positive BSA and/or PSA on the right side of the abdomen (58.3%, 35/60). Of the foreign body tests, positive results were seen with the back grip in 13.6% (8/59) of the cows, the pole test in 5.1% (3/59), percussion of the reticular area in 1.7% (1/59) and several of these tests in 13.6% (8/59). At least one foreign body test was positive in 33.9% (20/59) of the cows (Fig. 1; Table 1). On transrectal palpation, relatively empty small intestines were detected in 16.7% (10/60), taut bands of tissue suggesting a strangulation in 10.0% (6/60), a distended rumen in 15.0% (9/60) and various other abnormalities including an unidentified hollow organ and crepitus in 16.7% (10/60) of the cows. Twenty-four cows had more than one abnormal transrectal finding, and 11 had none. Faecal colour was dark to black in 10.0% (6/60) of the cows, and faecal consistency varied from liquid to pulpy to thick pulpy. Abnormal faecal contents included mucus (n = 13), blood (n = 10), fibrin (n = 4) and combinations thereof (n = 6).

### Other clinical findings

Other clinical abnormalities were reduced skin surface temperature (67.8%, 40/59), reduced skin turgor (51.7%, 31/60), prolonged capillary refill time (49.2%, 29/59), enophthalmus (48.3%, 29/60), moderate to severe scleral injection (46.6%, 27/58), foul or ammonia-like breath (26.7%, 16/60), dry cool muzzle (22.0%, 13/59), pale oral mucosa (8.5%, 5/59) and droopy ears (3.3%, 2/60).

### Urinalysis

The urine pH of 52 samples ranged from 5.0 to 9.0 (median, 8.0) and was acidic (5.0 to 6.9) in 34.6% (18/52) and alkaline (8.1 to 9.0) in 17.3% (9/52) of the cows. Specific gravity ranged from 1.002 to 1.060 (mean ± sd = 1.028 ± 13) and was < 1.020 in 25.5% (13/51) and > 1.040 in 13.8% (7/51) of the cows. Ketonuria (10 to > 150 mg/dL urine) occurred in 25.5% (13/51), haemoglobinuria/haematuria (5 to 250 erythrocytes/μl urine) in 24.0% (12/50), glucosuria (50-1000 mg/dL urine) in 19.6% (10/51) and proteinuria (100 to 500 mg protein/dL urine) in 3.9% (2/51) of the cows.

### Laboratory findings

**Fig. 2:**
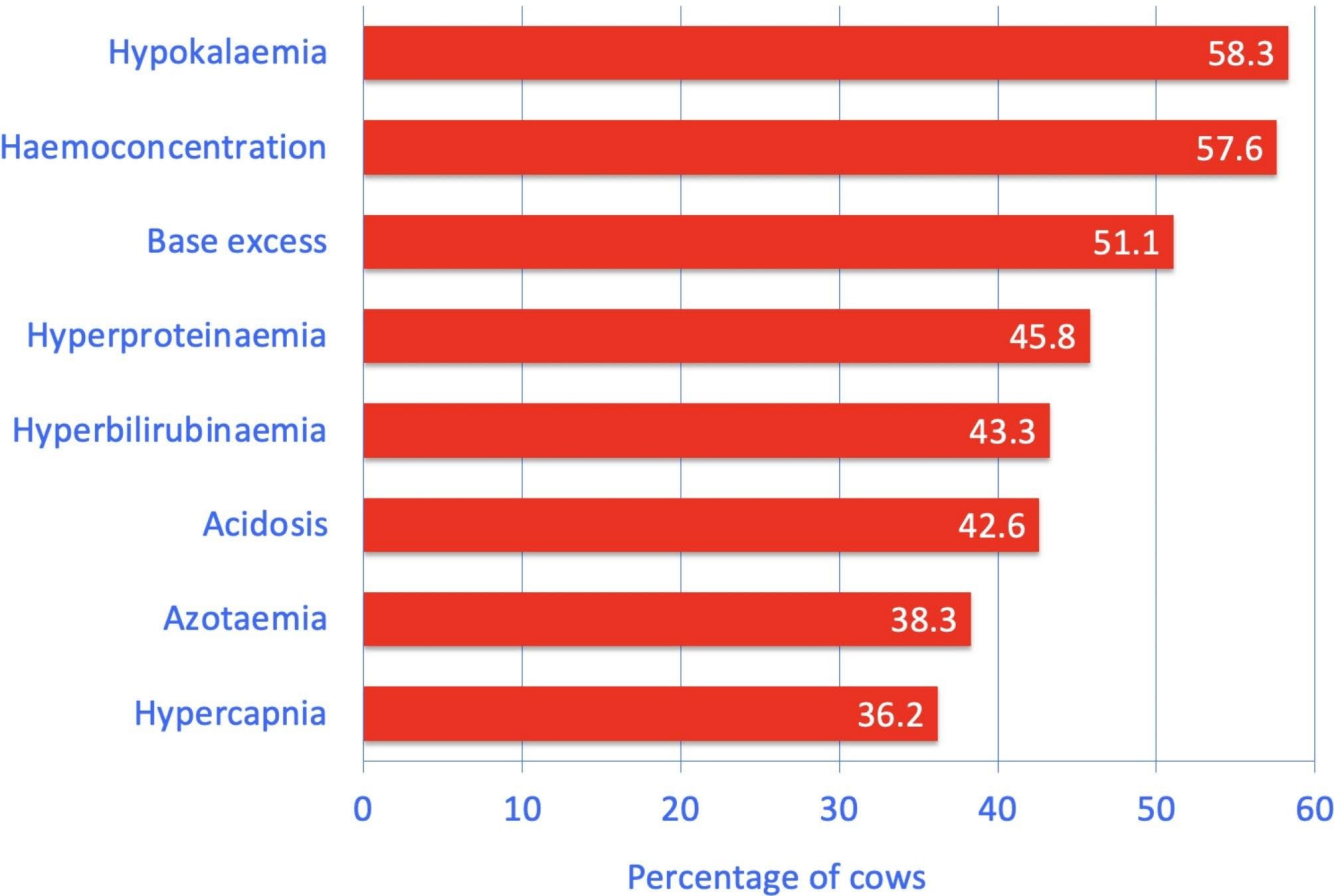
The most common abnormal blood variables in 60 cows with small intestinal strangulation. Acidosis was defined as a pH below 7.41

The major abnormalities were hypokalaemia (58.3%, 35/60), haemoconcentration (57.6%, 34/59), base excess (51.1%, 24/47), hyperproteinaemia (45.8%, 27/59), hyperbilirubinaemia (43.3%, 26/60), acidosis (42.6%, 20/47), azotaemia (38.3%, 23/60) and hypercapnia (36.2%, 17/47) (Fig. 2; Table 2). Increased activity of AST (31.7%, 19/60), hyperchloraemia (30.0%, 18/60), hypochloraemia (30% 18/60), leukocytosis (27.1%, 16/59), hyperfibrinogenaemia (25.9%, 15/58), increased bicarbonate (25.5%, 12/47), alkalosis (25.5%, 12/47) and base deficit (19.1%, 9/47) occurred less commonly. Rumen chloride was increased in 30.8% (16/52) of the cows.

**Table 2:** Laboratory findings in cattle with small intestinal strangulation (means ± standard deviations, medians with 25th to 75th percentiles, frequency distributions)

Variable (Mean ± sd, Median with 25th to 75th percentiles)	Finding	Number of cows	Percent
Haematocrit (n = 59) (36.8 ± 5.4%)	Normal (30–35%) Decreased (25–29%) Increased (36–54%)	22 3 34	37.3 5.1 57.6
Total leukocyte count (n = 59) (8,700, 6,600 − 10,400/μL)	Normal (5,000–10,000/μL) Decreased (3,700-4,999/μL) Increased (10,001–18,300/μL)	39 4 16	66.1 6.8 27.1
Total protein (n = 59) (80.0, 73–84 g/L)	Normal (60–80 g/L) Increased (81–118 g/L)	32 27	54.2 45.8
Fibrinogen (n = 58) (6.0, 4–8 g/L)	Normal (4–7 g/L) Decreased (2.0-3.9 g/L) Increased (7.1–13.0 g/L)	40 3 15	68.9 5.2 25.9
Urea (n = 60) (5.6, 4.6–8.3 mmol/L)	Normal (2.7–6.5 mmol/L) Increased (6.6–42.9 mmol/L)	37 23	61.7 38.3
Bilirubin (n = 60) (6.0, 3.9–9.3 μmol/L)	Normal (0.5–6.5 μmol/L) Increased (6.6–34.0 μmol/L)	34 26	56.7 43.3
Calcium (n = 29) (2.17, 1.93–2.40 mmol/L)	Normal (2.30–2.60 mmol/L) Decreased (1.48–2.29 mmol/L) Increased (2.61–3.18 mmol/L)	8 19 3	26.7 63.3 10.0
Magnesium (n = 28) (1.07, 0.92–1.31 mmol/L)	Normal (0.80-1.00 mmol/L) Decreased (0.65–0.79 mmol/L) Increased (1.01–2.82 mmol/L)	7 4 18	24.1 13.8 62.1
Inorganic phosphate (n = 28) (1.47, 1.18–1.98 mmol/L)	Normal (1.30–2.40 mmol/L) Decreased (0.35–1.29 mmol/L) Increased (2.41–4.81 mmol/L)	15 9 5	51.7 31.0 17.2
Chloride (n = 60) (101, 93–107 mmol/L)	Normal (96–105 mmol/L) Decreased (61–95 mmol/L) Increased (106–117 mmol/L)	24 18 18	40.0 30.0 30.0
Potassium (n = 60) (3.8 ± 0.8 mmol/L)	Normal (4.0–5.0 mmol/L) Decreased (2.0-3.9 mmol/L) Increased (5.1–5.8 mmol/L)	22 35 3	36.7 58.3 5.0
AST (n = 60) (84, 69–113 U/L)	Normal (50–103 U/L) Increased (104–656 U/L)	41 19	68.3 31.7
γ-GT (n = 60) (19, 15–23 U/L)	Normal (9–30 U/L) Increased (31–276 U/L)	52 8	86.7 13.3
pH (n = 47) (7.41 ± 0.07)	Normal (7.41–7.45) Decreased (7.20–7.40) Increased (7.46–7.58)	15 20 12	31.9 42.6 25.5
pCO_2_ (n = 47) (43.3, 39–51 mmHg)	Normal (35.0–45.0 mmHg) Decreased (29.8–34.9 mmHg) Increased (45.1–70.8 mmHg)	27 3 17	57.4 6.4 36.2
Bicarbonate (n = 47) (26.5, 22–32 mmol/l)	Normal (20.0–30.0 mmol/L) Decreased (15.1–19.9 mmol/L) Increased (30.1–59.6 mmol/L)	31 4 12	66.0 8.5 25.5
Base excess (n = 47) (2.4, -1.6- +5.0 mmol/L)	Normal (-2 - +2 mmol/L) Decreased (-8.6 - -2.1 mmol/L) Increased (+ 2.1 - +25 mmol/L)	14 9 24	29.8 19.1 51.1
Rumen chloride (n = 52) (25, 18–33 mmol/L)	Normal (≤ 30 mmol/L) Increased (31–101 mmol/L)	36 16	69.2 30.8

**Fig. 3:**
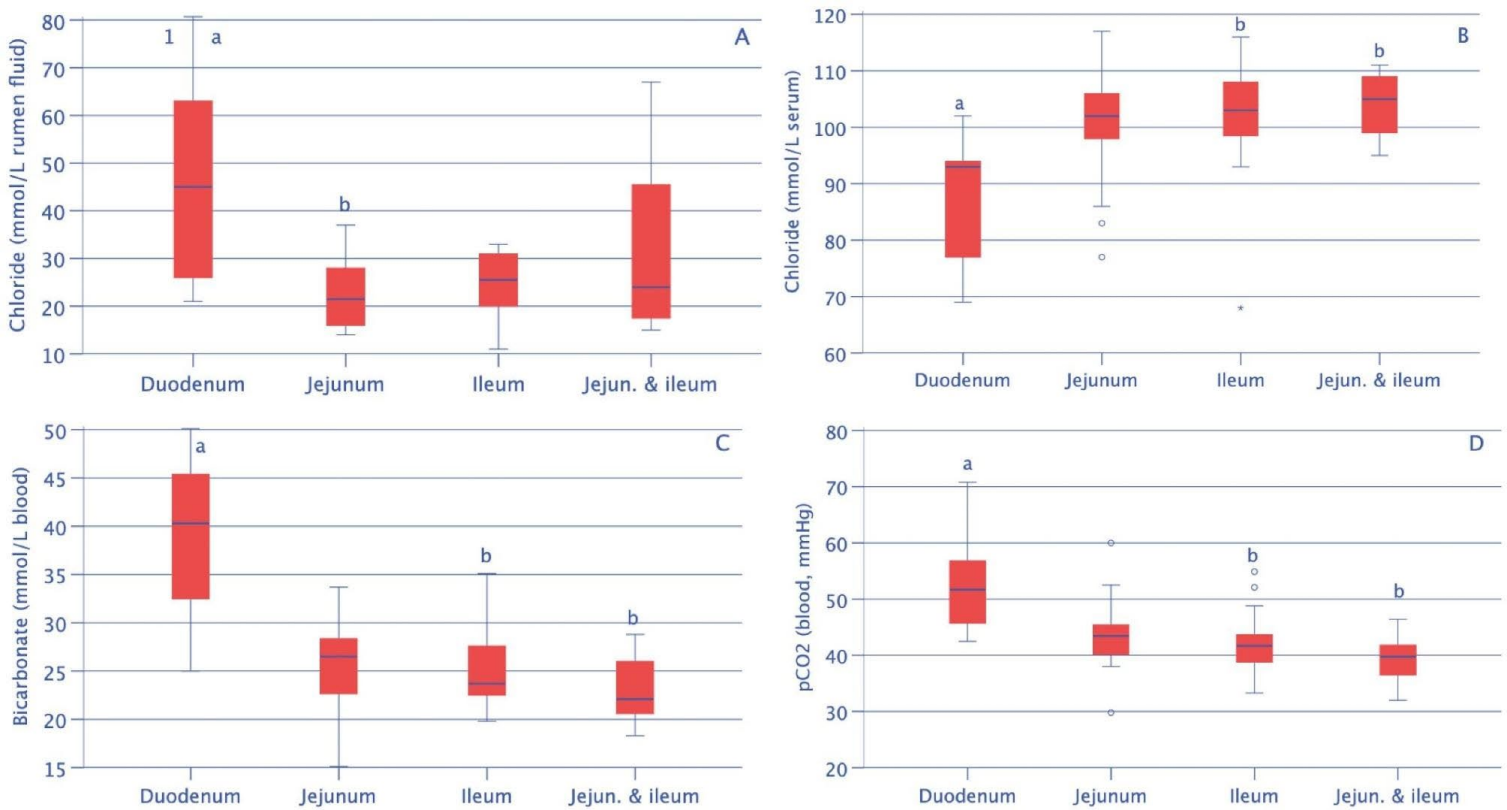
Concentrations of rumen chloride (**A**), serum chloride (**B**), bicarbonate (**C**) and pCO_2_ (**D**) depending on the anatomic location of the ileus. Boxplot presentation as described by Field [37]: Within a box, the thick horizontal line shows the median. The top and bottom of the blue box represent the upper and lower quartiles, respectively. The distance between the top of the box and the top of the whisker shows the range of the top 25% of scores. Similarly, the distance between the bottom of the box and the end of the bottom whisker shows the range of the lowest 25% of scores. ° = Outlier, ^*^ Extreme score. Within rows, values with different superscripts differ (P < 0.05). ^1^ Top of the whisker = 101 mmol/L

**Fig. 4:**
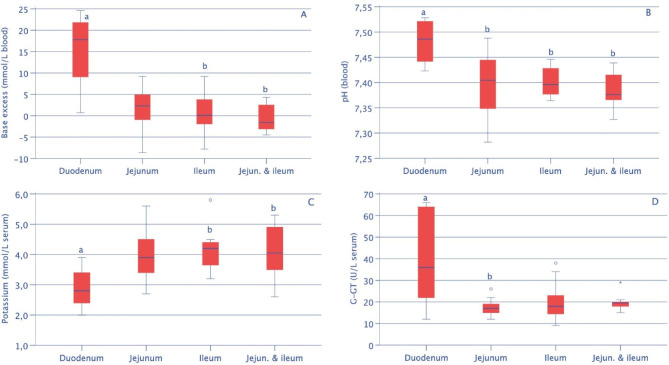
Base excess (**A**), pH (**B**), potassium concentration (**C**) and γ-GT activity (**D**) depending on the anatomic location of the ileus. See Fig. 3 for boxplot presentation and key

Several laboratory variables (Table 3) varied significantly depending on the anatomic location of the obstruction: the median rumen chloride concentration was highest in cows with duodenal obstruction (45 mmol/L) compared with cows with jejunal (22 mmol/L) or ileal obstruction (26 mmol/L) (Fig. 3A), whereas the median serum chloride concentration was lowest in cows with duodenal obstruction (93 vs. 102 and 103 mmol/L) (Fig. 3B). As a result of compensation mechanisms, cows with duodenal obstruction also had the highest median bicarbonate concentration (40 vs. 27 and 24 mmol/L) (Fig. 3C), highest pCO_2_ (52 vs. 44 and 42 mmHg) (Fig. 3D), highest base excess (18 vs. 2 and 0 mmol/L) (Fig. 4A) and highest blood pH (7.49 vs. 7.40 and 7.40) (Fig. 4B) and the lowest serum potassium concentration (2.8 vs. 3.9 and 4.2 mmol/L) (Fig. 4C). Cows with duodenal obstruction also had the highest γ-GT (36 vs. 17 and 18 U/L) (Fig. 4D) and AST activities (145 vs. 83 and 69 U/L) and the highest bilirubin concentrations (11 vs. 6 and 5 μmol/L).

**Table 3:** Laboratory findings in cattle with small intestinal strangulation depending on anatomic location of the ileus (means, medians, standard deviations, 25th to 75th percentiles)^1^

Variable	Finding	Duodenum	Jejunum	Ileum	Jej + Il
Rumen chloride	Mean/Median	50/45^a^	23/22^b^	24/26	32/24
(mmol/L)	SD (25th-75th percentiles)	29 (21–26)	7 (14–16)	8 (11–14)	19 (15–17)
	n	9	18	14	8
Potassium	Mean/Median	2.9/2.8^a^	3.9/3.9	4.1/4.2^b^	4.0/4.1^b^
(mmol/L)	SD (25th-75th percentiles)	0.6 (2.0-2.3)	0.8 (2.7–3.2)	0.6 (3.2–3.5)	0.8 (2.6–3.2)
	n	10	21	16	10
Chloride (mmol/L)	Mean/Median	87/93^a^	101/102	102/103^b^	104/105^b^
	SD (25th-75th percentiles)	11(69–75)	10 (77–88)	11 (93–98)	6 (95–100)
	n	10	21	16	10
pH	Mean/Median	7.48/7.49^a^	7.40/7.40^b^	7.39/7.40^b^	7.39/7.38^b^
	SD (25th-75th percentiles)	0.04 (7.42–7.44)	0.06 (7.28–7.35)	0.06 (7.36–7.38)	0.04 (7.33–7.37)
	n	9	12	13	10
Base excess	Mean/Median	15/18^a^	2/2	1/0^b^	-1/-2^b^
(mmol/L)	SD (25th-75th percentiles)	9 (0.7–6.3)	5 (-8.6 - -1.6)	4 (-4.8- -1.9)	3 (-4.5- -4.0)
	n	9	12	13	10
Bicarbonate	Mean/Median	38/40^a^	26/27	25/24^b^	23/22^b^
(mmol/L)	SD (25th-75th percentiles)	9 (25–30)	5 (15–22)	4 (20–23)	3 (18–20)
	n	9	12	13	10
pCO_2_	Mean/Median	53/52^a^	44/44	43/42^b^	40/40^b^
(mmHg)	SD (25th-75th percentiles)	9 (43–45)	7 (30–39)	6 (33–39)	4 (32–36)
	n	9	12	13	10
Bilirubin (μmol/L)	Mean/Median	14/11^a^	7/6	5/5^b^	6/6
	SD (25th-75th percentiles)	9 (5–7)	5 (3–4)	3 (1–3)	3 (2–3)
	n	10	21	16	10
AST (U/L)	Mean/Median	232/145^a^	108/83	78/69^b^	90/87
	SD (25th-75th percentiles)	217 (57–89)	70 (65–73)	31 (50–63)	23 (57–69)
	n	10	21	16	10
γ-GT (U/L)	Mean/Median	60/36^a^	17/17^b^	20/18	20/20
	SD (25th-75th percentiles)	78 (12–21)	3 (12–15)	8 (12–14)	4 (15–18)
	n	10	21	16	10

^1^ Of the variables listed in Table 2, only those with significant differences (Kruskal Wallis test) between locations are shown

Within rows, values with different superscripts differ (P < 0.05)

Jej + Il = Jejunum + Ileum

### Ultrasonographic findings

The principal findings were subjectively reduced or absent small intestinal motility (100%, 37/37) and dilated loops of small intestines (100.0%, 43/43) with a diameter of 3.6 to 10.2 cm and fluid between the loops (34.9%, 15/43) (Table 4). In 20.9% (9/43) of the cows, dilated loops were seen in juxtaposition with empty loops. The abomasum was dilated in 18.6% (8/43) of the cows because of retrograde accumulation of ingesta. The strangulation itself could not be visualised in any of the cows.

**Table 4:** Ultrasonographic findings in 43 cows with small intestinal strangulation

Variable	Finding		Number of cows	%
Intestinal motility (n = 37)	Subjectively decreased Absent		12 25	32.4 67.6
Cross-section of small intestines (n = 43)	Normal Dilated		0 43	0 100.0
Largest diameter of small intestines (n = 30) (median = 4.9 cm)	Normal (2.7–3.5 cm) Slightly dilated (3.6-4.0 cm) Moderately dilated (4.1-6.0 cm) Severely dilated (6.1–10.2 cm)		0 2 23 5	0 6.7 76.7 16.6
Empty poststenotic small intestines (n = 43)	Not visible Visible		34 9	79.1 20.9
Fluid between intestinal loops (n = 43)	No fluid visible Fluid without fibrin		28 15	65.1 34.9
Abomasum dilated (n = 43)	Not dilated Dilated		35 8	81.4 18.6

### Comorbidities

Fifty-one (85.0%) cows had no comorbidities identified. Clinical, intraoperative and/or postmortem examinations indicated 1 comorbidity in 11.7% (7/60) of the cows and multiple concomitant diseases in 3.3% (2/60), which included abomasal and duodenal ulcers, non-penetrating reticular foreign body, chronic peritonitis, chronic intraabdominal adhesions, bronchopneumonia and abscess.

### Parasitological faecal examination

Six of 25 faecal samples contained *Fasciola hepatica* eggs, which were accompanied by gastrointestinal nematode or *Dicrocoelium dendriticum* eggs in 5 cows. Seven other samples had only gastrointestinal nematodes, 3 had only *Dicrocoelium dendriticum* eggs and 4 had mixed infestation of these parasites. Five faecal samples contained no parasite eggs.

### Diagnoses

Based on the clinical findings, a *tentative diagnosis of ileus* was made in 26.7% (16/60) of the cows, a *diagnosis of ileus* was made in 48.3% (29/60) and a *diagnosis of ileus attributable to strangulation* was made in 10.0% (6/60) of the cows. In 15.0% (9/60) of the cows, no diagnosis was made or conditions such as caecal dilatation (4/60) or right displaced abomasum (1/60) were diagnosed.

Based on the ultrasonographic findings, a diagnosis of ileus was made in 86.0% (37/43) of the cows examined via ultrasonography; intestinal motility (37/37) was examined as well as the small intestines in cross-section (43/43). In 6 cows that were examined ultrasonographically, no diagnosis was made (n = 1) or other conditions such as dilatation of the abomasum (n = 1), duodenum (n = 1, colon (n = 1) or caecum (n = 2) were diagnosed.

A definitive diagnosis of strangulation was made in 100% (60/60) of cases based on the findings at laparotomy (n = 59) or postmortem examination (n = 1).

### Treatment and outcome

**Fig. 5:**
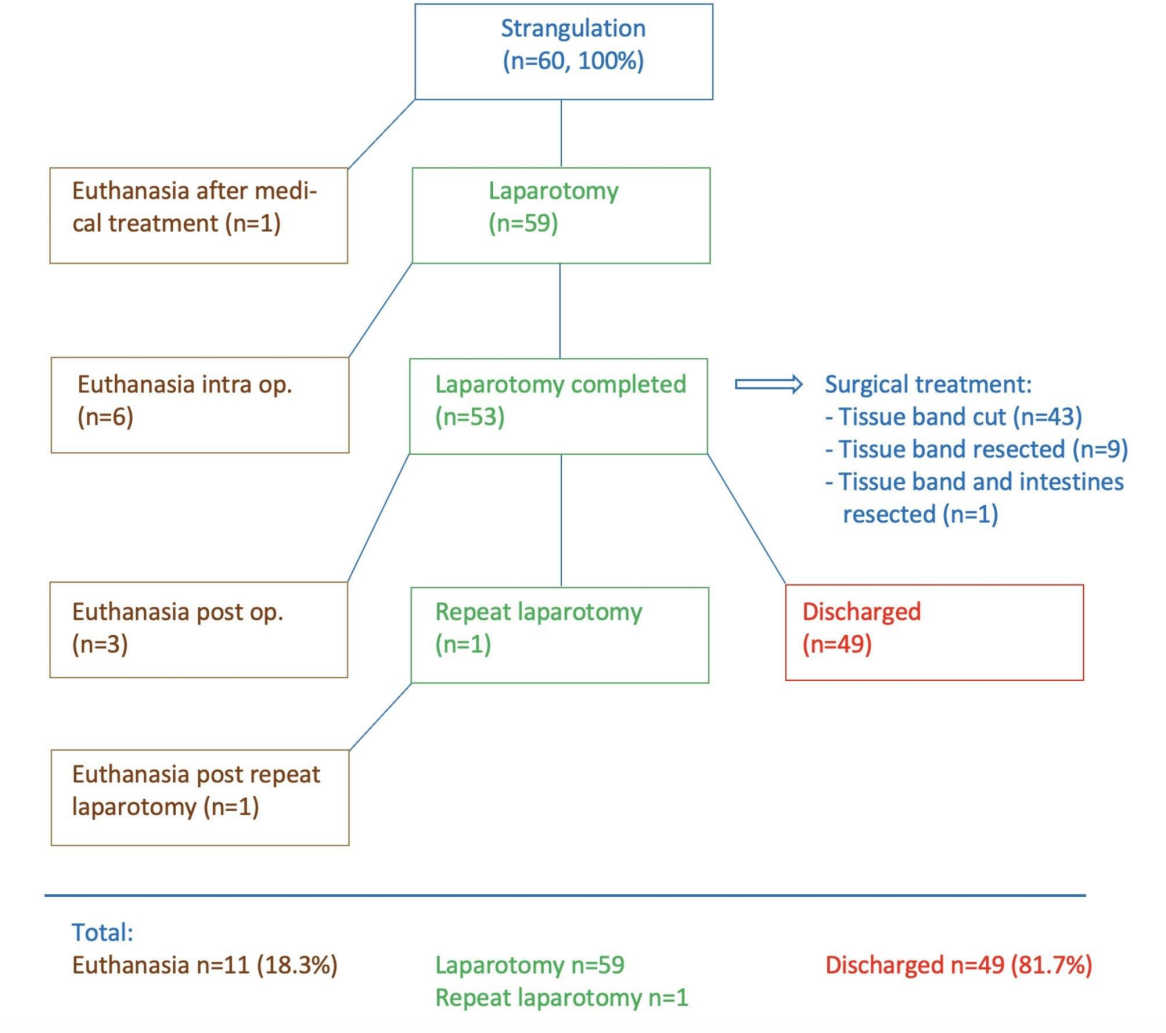
Treatment flowchart for 60 cattle with small intestinal strangulation

One cow with ambiguous findings was treated medically for 2 days, after which time she was euthanized because the owner declined surgical exploration of the abdomen (Fig. 5). Fifty-nine (98.3%) cows underwent right flank laparotomy immediately following the initial examination (57/60) or 1 or 2 days later (1/60). Six cows were euthanized intraoperatively. Of the 53 cows in which surgery was completed, the impinging tissue band was cut in 43 cows and resected in 10; one of the latter also underwent intestinal resection (resection of 80 cm of the jejunum, end-to-end anastomosis). Three of the operated cows were euthanized within 1 to 3 days of surgery, and another cow was euthanized one day after the second surgery due to intestinal paralysis. In summary, 98.3% (59/60) cows were operated, 18.3% (11/60) were euthanized before, during or after the first or second surgery and 81.7% (49/60) were discharged after successful treatment. Of the 43 cows in which the impinging tissue band was cut, 95.3% (41/43) were discharged and of the 9 that underwent resection of the tissue band, 88.9% (8/9) were discharged (P > 0.05, Fisher’s exact test).

### Surgical findings^1^ and intraoperative complications

The jejunum was most often involved in the strangulation (35.0%, 21/60), followed by the ileum (26.7%, 16/60) and duodenum (16.7%, 10/60). In 15.0% (9/60) of the cows, the jejunum and the ileum and in 1.6% (1/60), the jejunum, ileum and caecum were involved. The location of the strangulation was not noted in 5.0% (3/60) of the cows. The strangulation was caused by a single tissue band in 76.7% (46/60) of the cows, by two bands in 6.7% (4/60) and by three or more bands in 16.7% (10/60) of the cows.

The tissue band consisted of connective tissue in 40.0% (24/60) of the cases, a ligament in 18.3% (11/60), fibrin in 15.0% (9/60), a persistent umbilical artery in 1.7% (1/60) and a persistent urachus in 1.7% (1/60). In 23.3% (14/60) of the cows, the nature of the tissue band was not specified. Various abdominal and pelvic organs and structures were identified as origins and insertions of the tissue bands including omentum (26.7%, 16/60), mesentery (16.7%, 10/60), abdominal wall (13.3%, 8/60) and small intestines (13.3%, 8/60). In 25.0% (15/60) of the cows, the origin and the insertion of the tissue bands could not be identified or were not noted in the medical record. The intestines that were involved in the strangulation were normal in 45.0% (27/60) of the cows, mildly to moderately abnormal in another 45.0% (27/60) and severely abnormal in 10.0% (6/60).

Other intraoperative findings were adhesions (23.3%, 14/60), increased abdominal fluid (11.7%, 7/60) or other abnormalities including fibrin between the intestines (1/60), intestinal rupture (1/60), persistent umbilical vein (1/60) and nodular changes of the intestinal wall (1/60). In 51.7% (31/60) of the cows, no other changes were found in the abdomen. Eight point three per cent (5/60) of the 60 operated cows went down during surgery. Intestinal contamination did not occur, but one cow had bowel prolapse, which could not be replaced and therefore the cow had to be euthanized.

### Short-term outcome

The general condition of the 49 successfully treated cows normalised within 1 to 6 days, appetite returned to normal within 1 to 7 days and faecal output (with one exception) within 1 to 8 days (all medians = 2 days) after surgery. Between admission and 7 days postoperatively, the median rectal temperature ranged from 38.6 to 38.7 °C (P > 0.05) and the heart rate from 70 to 77 bpm (P > 0.05). These cows were discharged from the clinic in good health 2 to 15 days (median = 5 days) after surgery. Four cows deteriorated postoperatively and were euthanized 1 to 6 days (median = 2.5 days) after surgery.

### Long-term outcome

The long-term outcome was determined 2 years after discharge. Of the discharged cattle, 46.9% (23/49) had remained in the herd and were still productive, 1 cow had been slaughtered as a result of the original strangulation, 2 because of other illnesses and 11 for economic or unknown reasons. The long-term outcome of 12 cows was unknown.

### Postmortem findings

Eleven cows underwent postmortem examination. The principal findings in the small intestine were necrosis (7/60), haemorrhagic infarction (6/60), dilatation (2/60) and congestion (1/60) in the region of the strangulation. In two cows, severe proliferation of the greater omentum was seen. The strangulation was visible in one cow that was euthanized intraoperatively and in one cow that had received medical treatment. Intestinal rupture seen intraoperatively in one cow was confirmed. Two cows had non-perforating abomasal ulcers, which were accompanied by a duodenal ulcer in one cow. Another cow had a circular jejunal ulcer, conceivably attributable to a previous penetrating foreign body. A jejunal mucosal ulceration caused by the strangulation was seen in one cow, and another had a jejunal diverticulum at the site of the strangulation. Eight cows had peritonitis, two had enteritis and three cows had petechial haemorrhages in the intestines (2/60), heart (2/60), lungs (1/60), larynx (1/60), liver (1/60) and peritoneum (1/60). Multiple renal and liver infarcts, biliary fibrosis, hepatic lipidosis, oedema of the abomasum and colon, ruminal abscess, bronchopneumonia and oesophageal hyperkeratosis were seen in one cow each.

Based on the intraoperative, postmortem and/or parasitological findings, we surmised that the cause of the strangulation was peritonitis in 11 cows, fascioliasis in six (by liver flukes migrating through the peritoneal cavity from the small intestine to the liver), persistent urachus in one, persistent umbilical artery in one and an anomaly of unknown origin in one.

## Discussion

Only 40% (24/60) of cows had signs of visceral pain (colic) at the initial examination. This may have been related to the observation that signs of pain occur intermittently in cattle with SIS [21] and that phase 1 of ileus, the so-called *colic phase*, may only last from 2 to about 12 h, followed by the next phase, the *indolence stage* [22]. Signs of colic were not evident in the initial examination of several cattle with SIS [3–5, 10, 13]. In contrast, three cases had a history of abdominal pain [6, 10, 14]. This was in agreement with our results, in which more cows had a history of colic (53.3%, 32/60) than actual signs of colic at the initial examination (40.0%, 24/60). Most colicky cows had only one sign of abdominal pain, most commonly a sunken back (25.0%, 15/60). Interestingly, this was also the most common sign of pain (26.2%, 33/126) in cows with intussusception [17]. Shifting of weight from one hind foot to the other was the predominant sign of pain (28.6%, 18/63) in 63 cows with haemorrhagic bowel syndrome [23]. A sunken back and shifting of weight are relatively mild clinical signs, which may go unnoticed or be misinterpreted resulting in misjudgement of the overall condition. Abdominal guarding, which typically occurs in cows with somatic pain, was observed in almost half of all cows (48.3%, 29/60) with SIS. Somatic pain originates in the parietal peritoneum, omentum or root of the mesentery [24]. Abdominal guarding with a tense abdominal wall was also seen in cows with experimental intestinal ligation; the signs persisted until the cows died or the ligation was removed [25]. Abdominal guarding was also common (53.2%, 67/126) in cows with intussusception [17]. Non-specific signs of pain such as twitching of the anconeus muscle, bruxism and spontaneous grunting were less common and occurred in 23.3% (14/60) of the cows. The latter signs cannot be assigned to a specific organ or clinical condition but present a clear indication that the cow is ill and further examination is warranted.

A decreased rectal temperature was recorded in all cows (18/18) with experimental ligation of the small intestines [25], but only in about a third (31.7%, 19/60) of the cows of the present study. A lower-than-normal temperature was measured in 34.8% (8/23) of the cows with duodenal ileus [26], in 61.6% (77/125) of cows with intussusception [17] and in 73.0% (46/63) of cows with haemorrhagic bowel syndrome [23]. Reasons for decreased rectal temperature in cows with mechanical obstruction of the small intestine include dehydration and hypocalcaemia, but an empty rectum can lead to a spurious result. The different frequencies of a decrease in rectal temperature reflect the different levels of severity of the various diseases. Tachycardia was seen in 36.7% (22/60) of the cows of the present study, similar to 38.1% (48/126) of cows with intussusception [17] and 39.1% (9/23) of cows with duodenal ileus [26], whereas tachycardia occurred in 69.8% (44/63) of cows with haemorrhagic bowel syndrome [23]. The explanation for these differences is analogous to that given for a decrease in rectal temperature.

Changes in abdominal contour may be caused by retrograde accumulation of ingesta [5, 10]. The rumen of sick cows often has reduced fill because of poor appetite or anorexia but it can become overloaded in cows with proximal ileus [14, 26]. Thirteen (21.7%) cows had bilateral abdominal distension, which was accompanied by rumen overload in 6 cows. Reduction in ruminal motility (50.0%, 30/60) or rumen atony (43.4%, 26/60) were considered non-specific clinical signs caused by inhibitory inputs related to fever or pain acting on the gastric centre in the medulla oblongata [27]. By comparison, only 5.9% (29/489) of cows with traumatic reticuloperitonitis had rumen atony [28]. Rumen atony occurs primarily in cattle with severe illness, for instance in cows with toxic mastitis (57.0%, 90/158) [29] or type 2 abomasal ulcer (44.4%, 64/144) [30].

BSA and PSA were positive on the right side in 58.3% (35/60) of the cows. Both findings were considered important indications of ileus resulting from intestines distended by gas and/or fluid [31]. Dilated loops of small intestines could be palpated transrectally in 63.3% (38/60) of cows with strangulation compared with 24.6% (31/126) of cows with intussusception [17]; we assume that in some of the latter, the passage of intestinal contents was not completely abolished and the intestines were therefore less distended. A taut tissue band could be palpated transrectally in 10.0% (6/60) of the cows, but the actual SIS could not be detected. In addition to dilated intestinal loops, taut tissue bands could be palpated in a cow with a persistent vitello-umbilical ligament [3], in 1 of 2 cows with duodenal strangulation by the uterus [14] and in 2 cows with persistent urachal remnant [6, 10], but not in a cow with a ligament from the umbilicus to the liver [4]. In contrast to cows with SIS, the intussusception could be palpated in 22.9% (11/48) of cows [32], in 65% (13/20) of cows [33] and in 1.6% (2/126) of cows [17]. Faecal output was reduced in 38.3% (23/60) of the cows with SIS, and in 60.0% (36/60), the rectum was empty. Faecal consistency was abnormal in half of the cows that passed manure; as described for cows with experimental ligation of the small intestine [25], a thick pulpy consistency was most common, presumably because of a prolonged transit time through the large colon combined with dehydration. In 55% (33/60) of the cows, the rectum contained mucus, blood and/or fibrin. Thick tenacious mucus mixed with blood in the rectum always suggests mechanical blockage of the small intestine [34].

The haematocrit and total protein concentration were increased in 57.6% (34/59) and 45.8% 27/59) of the cows, respectively, and there was considerable overlap between these two findings suggesting that haemoconcentration attributable to dehydration was the underlying cause. This caused prerenal azotaemia with increased serum urea concentration in 38.3% (23/60) of the cows. Cows with intussusception had a slightly higher haematocrit (61.1%, 77/126) and total protein concentration (51.6%, 65/126), which could explain the higher rate of prerenal azotaemia (62.1%, 77/124) [17]. There was a striking relationship between the anatomic location of the strangulation and the rumen chloride, serum chloride, potassium and bilirubin concentrations, blood gas variables and enzyme activities. Except for the bilirubin concentration and enzyme activities, these relationships can be explained by the fact that duodenal ileus, because of its more proximal location, results in a more pronounced abomasal reflux syndrome associated with hypokalaemic metabolic alkalosis compared with ileus of the jejunum or ileum [35]. The bilirubin concentration and the activity of γ-GT are increased with duodenal ileus when transport of bile through the bile duct into the duodenum is restricted. An analogous explanation applies to cows with abomasal torsion, in which the activity of γ-GT is increased because of biliary obstruction as a result of displacement and distortion of the duodenum [36].

Dilated loops of small intestines and reduced or absent intestinal motility were the principal ultrasonographic findings, which are typical of ileus [11], but not specific indicators of intestinal strangulation. The strangulation itself could not be visualised in any of the cows using transabdominal ultrasonography. It is conceivable that transrectal ultrasonography could have improved the diagnosis. After all, transrectal examination allowed the palpation of a tissue band and it is possible that this could have been visualised and characterised ultrasonographically. We recommend that cows with a tentative diagnosis of ileus, particularly those with abnormal transrectal palpation findings, be scanned transrectally. Additionally, ultrasonographic examination from the ventral abdominal wall is indicated to detect tissue bands originating from the umbilical region.

The clinical examination facilitated a diagnosis of ileus in 48.3% (29/60) of the cows and intestinal *strangulation* as the cause of the ileus in another 10% (6/60). Although a comprehensive ultrasonographic examination of intestinal motility and diameter was done in only 37 of the 60 cows, the variables *reduced or absent intestinal motility* and *dilated small intestines*, which are obligatory for a diagnosis of ileus, could be confirmed ultrasonographically in all 37 cows. This demonstrates clearly that abdominal ultrasonography facilitates the diagnosis of ileus. Thus, ultrasonography should be part of the examination of all cows with severe gastrointestinal disorders.

Surgical treatment with mechanical resolution of the strangulation is the treatment of choice for intestinal strangulation and a necessity for the survival of the cow [1, 2]. The only cow that received medical treatment died after 2.5 days,^2^ while 81.7% (49/60) of the operated cows were discharged in good health 2 to 15 days (median = 5 days) after surgery. Thus, the short-term outcome of cows with strangulation was considerably higher than that of cows with other types of intestinal obstruction including duodenal ileus (47.8%, 11/23) [26], intussusception (44%, 56/126) [17] and haemorrhagic bowel syndrome (30.2%, 19/63) [23]. Possible reasons for the improved outcome of cows with intestinal strangulation include the facts that the intestinal changes were less severe than in cows with intussusception and haemorrhagic bowel syndrome and that the primary disease process was extraintestinal. Therefore, the small intestines could resume their normal function after transection of the tissue causing the obstruction, provided that the intestinal changes were not too severe. However, the prognosis is much less favourable in cows with protracted illness associated with more severe intestinal damage. In 43 cows, surgical treatment consisted of mere transection of the tissue band, whereas in 10, the obstructing tissue was resected. One of the latter cows also underwent bowel resection and anastomosis. The feasibility of resection depends on whether the affected portion of the intestine can be exteriorised; tissue bands that are merely palpable can be transected but not resected. This means that in cases with multiple locations of tissue bands that cannot be exteriorised, some may be missed. Contrary to an earlier recommendation [1] that resection is preferred to mere transection, the former method does not appear to result in a significant improvement in survival compared with the latter; in the present study, 95.3% (41/43) were discharged after transection and 88.9% (8/9) after resection of the obstructing lesion.

## Conclusions

Overall, intestinal strangulation is a rare cause of ileus and has a relatively good prognosis. Even though the site of the strangulation itself and the involved tissue bands could not be visualised, ultrasonography was useful for establishing a tentative diagnosis. As in all cases of ileus with a mechanical intestinal obstruction, immediate surgical treatment is of utmost importance for cow survival.

## Data Availability

The datasets used and analysed for this study are available from the corresponding author on reasonable request.

## Abbreviations

γ-GT: γ-Glutamyl transferase
AST: Aspartate aminotransferase
Bpm: Beats per minute
BSA: Ballottement and simultaneous auscultation
dL: Deciliter
EDTA: Ethylenediaminetetraacetic acid
Fig: Figure
g: Gram
IU: International units
Jej + Il: Jejunum + ileum
kg: Kilogram
L: Litre
mg: Milligram
Min: Minute
n: Number
pCO_2_: Partial pressure of carbon dioxide
PSA: Percussion and simultaneous auscultation
SD: Standard deviation
SIS: Small intestinal strangulation

## Footnotes

1 the postmortem findings from the non-operated cows are included.

2 The owner of this cow did not consent to surgery and because functional stenosis could not be ruled out, medical treatment was attempted.
